# Editorial: Advances in predisposition to bone marrow failure and hematopoietic neoplasms

**DOI:** 10.3389/fonc.2024.1377974

**Published:** 2024-02-16

**Authors:** Sushree S. Sahoo, Sherif Abdelhamed, Makiko Mochizuki-Kashio, Lara Wahlster

**Affiliations:** ^1^ Department of Hematology, St. Jude Childrens Research Hospital, Memphis, TN, United States; ^2^ Department of Pathology, St. Jude Childrens Research Hospital, Memphis, TN, United States; ^3^ Department of Mieroscopic and Developmental Anatomy, Tokyo Womens Medical University, Tokyo, Japan; ^4^ Department of Pediatric Oncology, Dana-Farber Cancer Institute, Boston, MA, United States; ^5^ Department of Hematology, Boston Childrens Hospital, Harvard Medical School, Boston, MA, United States

**Keywords:** germline predisposition, bone marrow failure, Hematopoietic neoplasms, diagnosis, genetics, therapy, pathomechanism

## 1 Introduction

Recent advances in genomic techniques have increasingly associated germline predisposition to hematopoietic malignancies (1). Inherited bone marrow failure syndromes (IBMFS) including Fanconi anemia (FA), Diamond-Blackfan anemia (DBA), Schwachman Diamond syndrome (SDS), and telomere biology disorders (TBD) are recognized as distinct hereditary blood disorders associated with a higher risk of developing a hematologic neoplasm. This list also includes the newly characterized group of myeloid neoplasms with bone marrow failure (BMF), caused by mutations in *GATA2*, *CEBPA*, *DDX41*, *RUNX1*, *ANKDR26*, *ETV6*, *SAMD9*, *SAMD9L*, and *ERCC6L2* (2–4). The progression from BMF to malignancy is a continuum influenced by both germline and additional acquired genetic events (5). Extensive studies on both pediatric and adult patient cohorts have illustrated certain somatic alterations to be associated with unfavorable clinical outcomes and decreased overall survival, underscoring the importance of early detection for effective patient management and therapeutic intervention (6–12). Once malignant transformation has occurred, hematopoietic stem cell transplantation (HSCT) often represents the only curative approach specifically in the setting of a preexisting germline predisposition, and presents with many challenges including of donor selection, conditioning regimen related toxicities, infections and post-HSCT complications (13, 14). Hence to augment the current treatment strategies, ongoing research efforts are focused on delineating the molecular dysregulation associated with BMF and leukemia progression to inform novel druggable pathways (15). Additionally, novel gene-editing approaches for gene correction are being explored as potential strategies to enable early interception prior to development of a hematologic neoplasm (16, 17).

**Figure 1:**
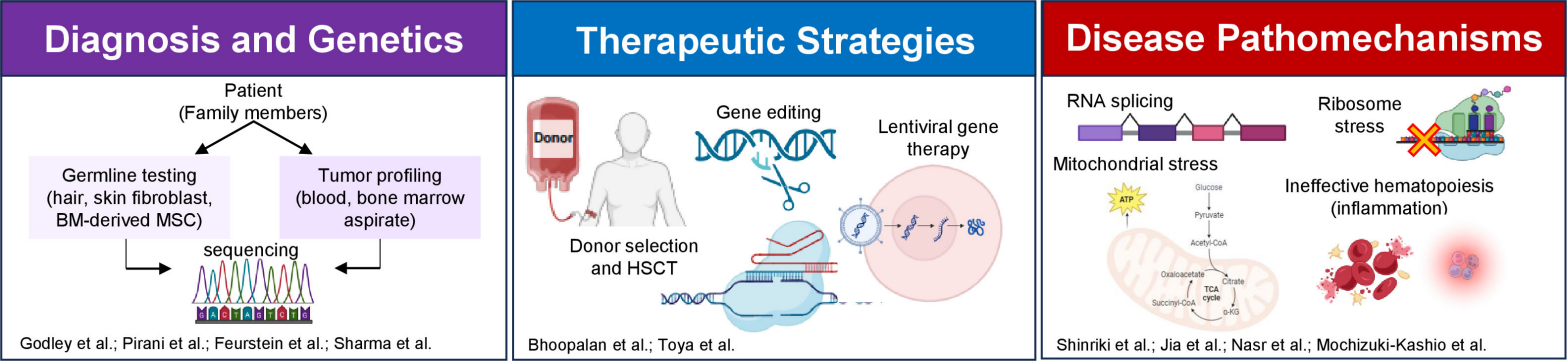
An overview of the Research Topic articles focusing on diagnosis, genetics, therapy and pathomechanism of predisposition to bone marrow failure and hematopoietic neoplasms. The figure is created using BioRender. BM, bone marrow; MSC, mesenchymal stem cells; HSCT, hematopoietic stem cell transplantation.

The Research Topic titled “*Advances in Predisposition to Bone Marrow Failure and Hematopoietic Neoplasms*” covers many aspects of this increasingly appreciated clinical and basic scientific field (**Figure 1**). This editorial serves as a spotlight to encompass these contributions, aiming to inspire further advancements and collaboration in the field.

## 2 Composition

Based on the scientific focus, 10 carefully selected articles were published as part of this Research Topic collection and are categorized to the following:

### 2.1 Diagnosis

With the interplay of germline and somatic genetics being increasingly recognized as a critical factor for the biology of various hematopoietic malignancies, adequate detection of both is critical. Towards that, in a review, Godley described the combination of germline genetic testing with tumor-based profiling in blood cancers for accurate risk assessment and treatment planning. The original research article by Perani et al. complimentarily highlights the importance of using appropriate biological samples to confirm germline variants, to enable adequate treatment planning early on. They compared sequencing data from bone marrow, blood, saliva, skin fibroblasts and hair follicles in a group of 29 patients and 44 relatives. The study revealed limitations in saliva testing due to tumor cell infiltration, while hair follicle DNA extraction showed potential as an alternative to skin biopsy.

### 2.2 Genetics

Over 100 genes have been associated with inherited BMF, and continued research is needed to fully understand their role in disease development and progression. Feurstein conducted a comprehensive review on four recently identified BMF syndromes involving genes *ERCC6L2*, *MECOM*, *DNAJC21*, and *ADH5/ALDH2*. Additionally, a new gene, called Replication protein A1 (*RPA1*) has recently been identified to cause TBD. In a brief research report, Sharma et al. found significant enrichment of novel and ultra-rare germline RPA1 variants in solid tumors, brain cancer, and hematological malignancies, suggesting a potential link between *RPA1* variants and predisposition to pediatric cancer.

### 2.3 Therapy

Treating BMF and hematopoietic malignancies that arise in the background of an underlying germline predisposition syndrome remains a complex and evolving topic. The mini-review by Bhoopalan et al. discusses important considerations for DBA patients and caregivers when deciding on HSCT. Factors like age, transfusion dependence, steroid response, and iron overload should be considered for eligibility. Gene correction by lentiviral vectors with *GATA1* gene overexpression or CRISPR/Cas9are promising alternative strategies. However, practical obstacles like limited access to stem cells, long-term efficacy and safety need to be addressed through further research and clinical trials. The second mini-review by Toya et al. specifically delves into adult-onset hereditary myeloid malignancies (HMM). The review highlights the challenges related to diagnosis, optimal treatment strategies, uncertainties regarding the timing and indication for HSCT, the risk of donor cell leukemia, and the absence of a recommended conditioning regimen for HMMs, emphasizing the need for further research to improve patient management in this area.

### 2.4 Pathomechanism

An important aspect of this Research Topic is exploring dysregulated molecular pathways in BMF and blood cancers for potential targeted therapy. This topic is covered by three systematic reviews and one original research article. The first review by Shinriki and Matsui provides an overview of *DDX41* mutations, which are found in approximately 2-5% of acute myeloid leukemia and myelodysplastic syndrome (MDS) patients. The review explores the role of DDX41 in various processes such as RNA splicing, DNA sensing in innate immunity, R-loop regulation, ribosome biogenesis, and translation. It also highlights the need for alternative treatments utilizing synthetic lethality, particularly for elderly patients who have difficulty with traditional cytotoxic chemotherapy. The second review by Jia and Gu discusses *PAX5* gene alterations and their impact on B-cell acute lymphoblastic leukemia. The third review by Nasr and Filippi highlights the emerging role of mitochondria in the development of BMF and MDS, emphasizing the impact of abnormal mitochondrial metabolism, dynamics and reactive oxygen species on ineffective hematopoiesis. In the final article of this Research Topic, Mochizuki-Kashio et al. conducted an original research exploring the effects of replication stress (RS) on mitochondrial function in a *Fancd2*-deficient FA mice model. The study revealed RS to affect mitochondrial activity and mitophagy in *Fancd2*-deficient fetal hematopoietic stem cells (HSCs) and adult bone marrow HSCs, pointing to mitochondrial metabolism defect in FA pathophysiology.

## 3 Conclusions and perspectives

The risk of malignancy in both inherited and acquired BMF disorders is shaped by disease specific genetics and associated cellular changes. This Research Topic has significantly raised awareness of the current advances in the diagnosis, molecular understanding and development of therapeutic strategies of BMF syndromes. Although progress has been made in identifying modifiable risk factors and developing targeted therapies, there is still significant work to do. To advance diagnosis, treatment, and patient outcomes in terms of overall survival and quality of life, it is crucial to integrate basic research with “omics” studies conducted on well-annotated clinical samples. This collaborative approach will pave the way for improvements in managing BMF and delivering better care to patients.

## Author contributions

SS: Writing – original draft, Writing – review & editing. SA: Writing – review & editing. MM-K: Writing – review & editing. LW: Writing – review & editing.
